# Multiple primary malignant neoplasms of the glottis, renal pelvis, urinary bladder, oral floor, prostate, and esophagus in a Japanese male patient: a case report

**DOI:** 10.1186/1477-7819-12-294

**Published:** 2014-09-22

**Authors:** Yoshihiro Mukaiyama, Motofumi Suzuki, Teppei Morikawa, Yoshiyuki Mori, Yuta Takeshima, Tetsuya Fujimura, Hiroshi Fukuhara, Tohru Nakagawa, Hiroaki Nishimatsu, Haruki Kume, Yukio Homma

**Affiliations:** Department of Urology, Saitama Medical Center, 4-9-3 Kitaurawa, Urawa-ku, Saitama, Saitama 330-0074 Japan; Department of Urology, Tokyo Teishin Hospital, 2-14-23 Fujimi, Chiyoda-ku, Tokyo 102-8798 Japan; Department of Urology, Graduate School of Medicine, The University of Tokyo, 7-3-1 Hongo, Bunkyo-ku, Tokyo 113-8655 Japan; Department of Pathology, Graduate School of Medicine, The University of Tokyo, 7-3-1 Hongo, Bunkyo-ku, Tokyo 113-8655 Japan; Department of Oral-Maxillofacial Surgery, Dentistry and Orthodontics, Jichi Medical University Hospital, 3311-1 Yakushiji, Shimotsuke, Tochigi 329-0498 Japan; Department of Urology, The Fraternity Memorial Hospital, 2-1-11 Yokoami, Sumida-ku, Tokyo 130-8587 Japan

**Keywords:** Metachronous, Multiple primary malignant neoplasms, p53 staining, Synchronous

## Abstract

Owing to recent advances in diagnostic and surgical techniques for cancer, a patient diagnosed with two or more neoplasms is not rare. We report on the case of a 58-year-old male with multiple primary malignant neoplasms, who suffered from three histological types of malignant neoplasm in six organs, namely the glottis, renal pelvis, urinary bladder, oral floor, prostate, and esophagus in chronological order. The first neoplasm was a squamous cell carcinoma of the glottis diagnosed in 2006. The second and third neoplasms were urothelial carcinomas of the right renal pelvis and urinary bladder, respectively, diagnosed in 2008. The remaining three neoplasms were diagnosed in 2010, namely a squamous cell carcinoma of the oral floor, an adenocarcinoma of the prostate, and a squamous cell carcinoma of the esophagus. The glottic cancer and esophageal cancer were treated by external radiation therapy. The malignant neoplasms of the oral floor and those which originated in the urinary tract were surgically resected. All neoplasms except the malignant neoplasm of the oral floor were well controlled. The patient died of cervical lymph node metastasis from the squamous cell carcinoma of the oral floor in January 2011. As far as we know, the present report is the first one on this combination of primary malignant neoplasms.

## Background

Early detection and development of novel treatment modalities are improving longevity of patients with cancer. Consequently, the increasing possibility of suffering from multiple primary malignant neoplasms (MPMN) is emerging as a common problem for cancer survivors; MPMN were first described by Billroth et al. [1]. In 1932, Warren and Gates proposed three criteria for the diagnosis of a second primary cancer: i) each tumor must present a definite clinical and histological picture of malignancy; ii) each tumor must be histologically distinct; and iii) the probability that one was a metastatic lesion from the other must be excluded [2]. Moertel proposed new definition of MPMN, where synchronous malignancies are those that occur within 6 months of the diagnosis of a previous malignant neoplasm and metachronous ones are those that occur more than 6 months apart [3]. According to the Surveillance, Epidemiology, and End Results (SEER) Program Coding and Staging Manual 2004, synchronous tumors are multiple tumors diagnosed within 2 months of the original/initial diagnosis, and metachronous ones are multiple tumors or lesions that occur more than 2 months after the original/initial diagnosis [4].

In this report, we describe the rare case of a patient who suffered from three histological types of malignant neoplasm in six organs, namely a squamous cell carcinoma (SCC) of the glottis, oral floor, and esophagus, a urothelial carcinoma (UC) of the right renal pelvis and urinary bladder, and an adenocarcinoma (AC) of the prostate.

## Case presentation

A 58-year-old male, a manager of a tavern, habitual drinker (one bottle of beer a day for 36 years), and heavy smoker (20 cigarettes a day for 33 years), was referred to our department for asymptomatic gross hematuria on February 2008. Two years before, he had been diagnosed with a SCC of the glottis, pT1N0M0 (stage I) which was treated by 70 Gy of external radiation therapy at the University of Tokyo Hospital. Since November 2007, he had been diagnosed with alcoholic liver cirrhosis and had received conservative medical treatment at a nearby hospital. He did not have any family history of malignant neoplasms. An abdominal computed tomography (CT) showed right hydronephrosis and tumor of the right renal pelvis. A retrograde pyelography also supported the findings of the abdominal CT. We conducted cystoscopy prior to the surgery; however, no distinct bladder tumors could be seen. We diagnosed him with right renal pelvic tumor and performed right nephroureterectomy on April 2008. The pathological diagnosis was UC of the right renal pelvis, high-grade, pT3N0M0 (stage III) including carcinoma *in situ* in the renal pelvis. Adjuvant chemotherapy was not administered due to chronic liver dysfunction and thrombocytopenia. Except for alcoholic liver cirrhosis, no other medical conditions or treatment-induced immunosuppression were recorded. After the surgery, follow-up was conducted by a cystoscopy every 3 months and an abdominal CT scan every 6 months.

In September 2008, cystoscopy revealed multiple bladder tumors. He underwent transurethral resection of bladder tumor (TURBT) in October 2008. The pathological finding was UC of the urinary bladder, high-grade, pTisN0M0 (stage 0is). He received weekly intravesical instillation of bovine Bacille-Calmette Guérin (Connaught strain, 81 mg/once a week) for 8 weeks to prevent recurrence of the bladder tumor.

In October 2009, he underwent TURBT again for recurrent bladder cancer. The pathological finding was UC of the urinary bladder, high-grade, pTis. UC was also found at the prostatic urethra, high-grade, pTis. We recommended a radical cystectomy and urethrectomy with urinary diversion. On December 2009, he became aware of a mass beneath his tongue. We found a papillary superficial tumor on the left side of the oral floor. Upon consultation with the department of oral surgery, the tumor was resected in January 2010. The pathological finding was a well differentiated SCC of the oral floor, pT1N0M0 (stage I). Just 2 weeks after the oral surgery, we performed radical cystectomy and urethrectomy with an ileal conduit urinary diversion. The pathological findings were UC *in situ* involving the urethra and prostatic duct, high-grade, pTisN0M0 (stage 0is) and incidental AC of the prostate, Gleason score 3 + 3, pT1aN0M0 (stage IIa).

In March 2010, 6 weeks after resection of the tumor of the oral floor, a head and neck CT scan suggested a submental lymph node swelling. He underwent bilateral cervical lymphadenectomy and 17 lymph nodes were resected. As a result, only one submental lymph node was diagnosed as a metastasis of well differentiated SCC of the oral floor. On June 2010, he experienced dysphagia. During an upper gastrointestinal endoscopy, biopsies for erosive lesions at the upper and middle portions of the esophageal mucosa were performed. The pathological finding was a well differentiated SCC of the esophagus, pT1N0M0 (stage I) and external radiation therapy with total 68.4 Gy was performed. From July 2010 onwards, cervical lymph node metastasis grew rapidly and gradually caused a tracheal obstruction. He underwent tracheotomy on November 2010. After receiving palliative care, he died of cancer cachexia on January 2011. An autopsy was not performed.

**Figure 1:**
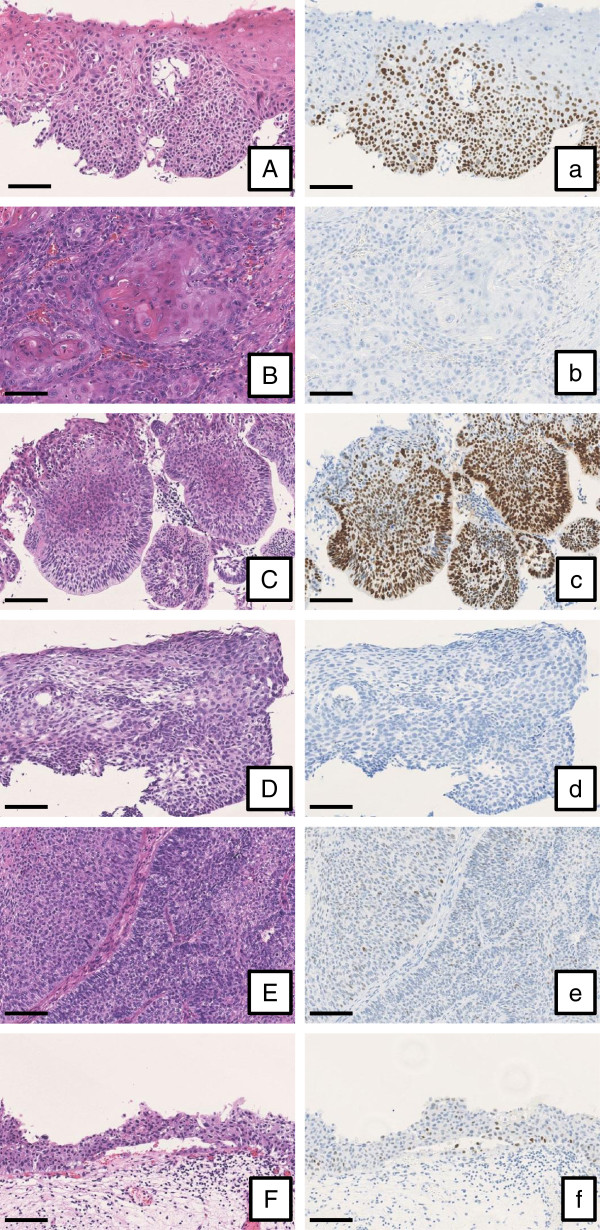
**The expression of p53 in SCC and UC.** Tissue samples were stained by hematoxylin-eosin (left column) and anti-p53 antibody diluted 1:50 (Clone DO-7, Leica Biosystems, Wetzlar, Germany; right column). Immunoreactivity of p53 in tumor cells was identified by nuclear brown color. **(A and** **a)** Glottic cancer; **(B** **and b)** Oral cancer; **(C and** **c)** Esophageal cancer (upper portion); **(D and** **d)** Esophageal cancer (middle portion); **(E and** **e)** Renal pelvic cancer; **(F and** **f)** Bladder cancer. Scale bars, 100 μm.

The malignant neoplasms in six organs are listed sequentially in Table 1. Figure 1 shows hematoxylin-eosin staining and immunohistochemical staining using anti-p53 antibody of SCC and UC. It is reported that nuclear immunoreactivity of p53 is a good surrogate of *TP53* mutations [5]. Immunoreactivity of p53 in tumor cells was different among SCCs; the glottic cancer and esophageal cancer in the upper portion were diffusely positive, while the oral cancer, esophageal cancer in the middle portion, and cervical lymph node metastasis of the oral cancer were completely negative. As for the renal pelvic cancer and bladder cancer, immunoreactivity of p53 was weakly positive in scattered cancer cells.

**Table 1:** **List of three histological types of malignant neoplasm in six organs**

Month/Year	Organ	Histology	Immunoreactivity of p53	ICD-O-3		pT-stage
				Site code	Histology code	
April/2006	Glottis	SCC	Diffusely positive	C320	8071/31	pT1
April/2008	Renal pelvis	UC	Weakly positive	C659	8120/33	pT3
October/2008	Urinary bladder	UC	Weakly positive	C675	8120/32	pTa
				C676	8120/23	pTis
January/2010	Oral floor	SCC	Completely negative	C041	8071/31	pT1
January/2010	Prostate	AC	Not examined	C619	8140/32	pT1a
June/2010	Esophagus			C154	8070/31	pT1
	Upper: 22 cm from incisor	SCC	Diffusely positive			
	Middle: 30 cm from incisor	SCC	Completely negative			

We classified malignant neoplasms by the International Classification of Diseases for Oncology version 3 (ICD-O-3) [6]. The T-stage was based on the Union for International Cancer Control TNM classification of malignant tumors (7th Edition) [7].

SCC, Squamous cell carcinoma; UC, Urothelial carcinoma; AC, Adenocarcinoma.

## Discussion

The actual risk of developing MPMN in Japan is unknown since cancer registration systems have yet to be legislated in Japan. Demandante et al. reviewed the literature published from 1966 to 2000 and reported that the prevalence of MPMN varied from 0.734% to 11.7% [8]. Based on epidemiological data from the National Cancer Institute’s SEER Program in 2003, it is estimated that approximately 16% of new cancers reported to their registry represent a second- or high-order malignancy [9]. The Osaka Cancer Registry, one of the regional cancer registration systems in Japan, has been operating since December 1962 and has accumulated about one million cancer incidence data in Osaka prefecture [10]. Using the Osaka Cancer Registry data, Tabuchi et al. reported that 10-year cumulative risk of metachronous second primary cancer in Japanese male patients was 10.2% at 50 to 59 years of age, 16.2% at 60 to 69 years of age, and 21.8% at 70 to 79 years of age [11].

This is an extremely rare case of MPMN in terms of the number of malignant neoplasms and their combination. We could not find out any case reports of the same combination of neoplasms on either the PubMed or the Japan Medical Abstracts Society databases. It is difficult to estimate the risk of quintuple cancers; however, Rabbani et al. estimated the risk of MPMN in patients with renal cell carcinoma [12]. Of 551 patients, they reported that the incidence of double cancer was 26.9%, triple cancer was 6.2%, quadruple cancer was 1.1%, and quintuple cancer was 0.2% [12]. Liu et al. studied the types of second primary malignancies among the patients with head and neck cancer [13]. The types of second primary malignancy with adjusted hazard ratio (95% confidence interval) were esophageal cancer 3.47 (2.40 to 5.03), prostate cancer 0.94 (0.45 to 1.95), bladder cancer 0.90 (0.39 to 2.10), among others [13]. Powell et al. considered the prognosis of patients with MPMN [14] by dividing patients with MPMN into synchronous and metachronous cases according to the classification proposed by Moertel [3]. Overall survival ratio was significantly lower in the patients with synchronous MPMN than those with metachronous MPMN (adjusted hazard ratio 0.50, *P* <0.001).

Regarding the etiology of multiple primary malignancies, several factors have been incriminated such as genetic, hormonal (e.g., sex steroid), iatrogenic (e.g., chemotherapy, radiation therapy, hormonal and immunosuppressive medications), and immunologic factors [15, 16]. In particular, tobacco smoking causes cancers of the lung, oral cavity, naso-, oro-, and hypopharynx, nasal cavity and accessory sinuses, larynx, esophagus, stomach, pancreas, colorectum, liver, kidney (body and pelvis), ureter, urinary bladder, uterine cervix and ovary (mucinous), and myeloid leukemia [17]. In the present case, alcoholic liver cirrhosis, tobacco smoking, and external radiation therapy for glottic cancer may have played a role in MPMN development. Several investigators have observed that patients with cancers of these sites developed a second primary cancer within the same anatomic region [18–20]. Wynder et al. publicized that continuation of smoking habit after diagnosis of the index cancer increased the risk for development of a second primary lesion [21]. Heavy alcohol consumption also predisposes certain sites to tumorigenesis, but primarily in conjunction with tobacco usage. In spite of cessation of smoking and drinking habits after the diagnosis of the glottic cancer, our patient developed MPMN.

Hereditary forms of adult cancers are also frequently associated with multiple primary cancers [22, 23]. If the nature of the hereditary predisposition is similar to that of childhood cancers, i.e., an inherited mutation that reduces the subsequent number of mutations necessary in each cell of the target organ, then such individuals may have a high rate of spontaneous tumors and a unique sensitivity to environmental agents [24]. Kotnis et al. conducted a case control study to assess genetic predisposition in a biologically-enriched clinical model system of tobacco-related cancers occurring as MPMN. They found that tobacco habit and three genetic polymorphisms, including *Tp53* (Arg72Arg), *XRCC1* (Arg399His), and *meH* (Tyr113His), formed the best model for developing tobacco-associated MPMN [25].

In 1953, Slaghter et al. proposed field cancerization, a process whereby the epithelial lining has been continuously exposed to tobacco and/or alcohol, leading to extensive premalignant and malignant cytologic changes and an increased risk for multiple independent tumor development [26]. Field cancerization in the epithelium has become accepted as playing a role in the development of MPMN in the same system; however, molecular studies have supported an alternative theory of a common clonal origin [27]. These studies suggest that a proportion of second primary head and neck SCC is clonally related to an index tumor, despite the presence of intervening normal mucosa and significant separation by normal mucosa. The same types of carcinogens and oncogenes seem to be responsible for the development of neoplasms in cases of other associations such as breast and endometrial/ovarian tumors [28–31].

In the present case, immunoreactivity of p53 in tumor cells was different among SCCs, suggesting that these cancers were polyclonal, although distinct genetic abnormalities including loss of heterogeneity, gene mutations, and influence of oncogenic viral infection may be necessary to definitely determine polyclonality.

Another point of interest may be the origin of UC of the renal pelvis and urinary bladder. According to the report from the International Agency for Research on Cancer [32], high-grade UC is likely to have high level amplifications and *TP53* mutations. In the present patient, however, immunoreactivity of p53 in the both high-grade UCs was very weak. The atypical finding ironically suggests UC of the renal pelvis and urinary bladder might be of monoclonal origin.

Modern molecular biologic techniques might help in the understanding of the biology involved and in designing effective diagnostic and therapeutic strategies to deal with these tumors.

## Conclusions

A rare case of MPMN of three histological types of malignant neoplasm in six organs was reported. Epidemiological and clinicopathological studies of MPMN are crucial for early detection and proper intervention of high risk patients.

## Consent

The study was conducted with the approval of the Ethics Committee of the University of Tokyo. Written informed consent was obtained from the patient for publication of the case report.

## Abbreviations

AC: Adenocarcinoma
CT: Computed tomography
MPMN: Multiple primary malignant neoplasms
SCC: Squamous cell carcinoma
SEER: Surveillance, Epidemiology, and End Results
TURBT: Transurethral resection of bladder tumor
UC: Urothelial carcinoma.
